# Abscisic Acid Induces Triacylglycerol Accumulation and Lipid Remodelling in Chloroplast‐Containing Green Tissues of *Lemna minor*

**DOI:** 10.1111/pce.70386

**Published:** 2026-01-20

**Authors:** Eunbi Kim, Bae Young Choi, Sujeong Je, Joohyun Kang, Seungwoo Shin, Yoomi Roh, Min Kim, Shogo Ito, Tokitaka Oyama, Yuree Lee, Donghwan Shim, Yasuyo Yamaoka

**Affiliations:** ^1^ Division of Biotechnology The Catholic University of Korea Bucheon Republic of Korea; ^2^ School of Liberal Arts and Sciences Korea National University of Transportation Chungju Republic of Korea; ^3^ Research, Institute of Basic Sciences Seoul National University Seoul Republic of Korea; ^4^ School of Biological Sciences Seoul National University Seoul Republic of Korea; ^5^ Department of Botany, Graduate School of Science Kyoto University Kyoto Japan; ^6^ Research Center for Plant Plasticity Seoul National University Seoul Republic of Korea; ^7^ Department of Biological Sciences Chungnam National University Daejeon Republic of Korea; ^8^ Center for Genome Engineering Institute for Basic Science Daejeon Republic of Korea

**Keywords:** abscisic acid, duckweeds, fatty acid, lipid droplets, triacylglycerols

## Abstract

Lipid remodelling is a fundamental component of plant responses to environmental stress and development, yet its regulation in fast‐growing aquatic plants remains poorly understood. Here, we investigated how abscisic acid (ABA) regulates triacylglycerol (TAG) accumulation and fatty acid (FA) composition in the duckweed *Lemna minor*. A 3‐day treatment with 1 µM ABA induced a 2.9‐fold increase in TAG content, accompanied by extensive remodelling of plastidial and extraplastidial membrane lipids. Reduced monogalactosyldiacylglycerol (MGDG) likely served as a FA source for TAG synthesis. Transcript analyses revealed strong induction of *diacylglycerol acyltransferase* (*DGAT*) genes, catalysing the final step of TAG formation, and repression of *fatty acid desaturase* (*FAD*) genes, resulting in a marked reduction in polyunsaturated FA levels. Confocal imaging confirmed substantial lipid droplet accumulation in both fronds and chloroplast‐containing roots. Notably, this sustained ABA‐induced TAG accumulation was unique to *L. minor*, with no comparable response observed in other duckweed species or in *Arabidopsis* under identical treatment. These findings reveal a species‐specific ABA‐driven lipid remodelling pathway in duckweed, linking phytohormone signalling to carbon storage in aquatic plants.

## Summary statement

ABA induces a unique lipid remodelling pathway in *Lemna minor* under environmental signalling, leading to strong triacylglycerol accumulation through membrane lipid turnover, *DGAT* activation, and *FAD* repression. This species‐specific response highlights duckweed as a model for studying environment‐driven carbon storage in aquatic plants.

**Abbreviations**
ABA: abscisic acid
ACC: 1‐aminocyclopropane‐1‐carboxylate
FA: fatty acid
GA: giberellc acid
IAA: indole‐3‐acetic acid
JA: jasmonic acid
TAG: triacylglycerol
tZ.: trans‐zeatin

## 1 Introduction

Duckweed is a globally distributed free‐floating aquatic plant that belongs to a family of aquatic monocotyledonous plants comprising 36 recognised species across five genera: *Spirodela, Landoltia, Lemna, Wolffiella*, and *Wolffia* (Acosta et al. 2021; Sree et al. 2015). Duckweeds are among the fastest‐growing plants, with a doubling time ranging from 16 h to 3 days under optimal conditions (Leng et al. 1995; W. Wang et al. 2010). Their small size and rapid growth, coupled with their aquatic habitat, allow them to be cultivated without competing for arable land. Consistent with these traits, duckweeds exhibit highly acquisitive traits, including an extremely low leaf mass per area, short lifespan, and high mass‐based photosynthetic rate (*A*_mass_) (Ishizawa et al. 2021). These features suggest that duckweeds could serve as a valuable resource for bioenergy applications (Kang et al. 2025).

Enhancing the accumulation of beneficial compounds can increase the value of duckweed as both a food and energy source. Given their rapid growth and adaptability, duckweeds have attracted increasing attention as promising candidates for bioenergy production. Among the target metabolites, triacylglycerols (TAGs), which contain more than twice the energy density of starch (Liang et al. 2023), are major energy‐storage metabolites in plants. With the growing demand for sustainable biofuels, considerable efforts have been directed toward producing biodiesel and bioethanol from plant biomass. Given its potential for high TAG content, duckweed could serve as an attractive biofuel feedstock. However, our understanding of lipid metabolism in duckweed remains limited. Most studies to date have focused on measuring lipid content and composition, as well as evaluating duckweed's potential as a biofuel feedstock (G. Chen et al. 2022; Tang et al. 2015). Although recent efforts using genetic engineering have successfully enhanced TAG accumulation (Liang et al. 2023), the underlying metabolic and regulatory pathways remain largely unexplored. Gaining deeper insight into these pathways will be essential for the rational design of duckweed‐based bioenergy applications.

In order to effectively manipulate these metabolic pathways, it is essential to consider the role of internal regulatory mechanisms, particularly phytohormones. Phytohormones play a crucial role in plant growth, development, defense, and responses to environmental stresses. Cytokinin is an essential phytohormone that sustains cell division and promotes stem development in shoots (Kieber and Schaller 2014; Svolacchia and Sabatini 2023). It is also implicated in root development, particularly by promoting the differentiation of meristematic cells (Svolacchia and Sabatini 2023). Gibberellins (GAs) are vital phytohormones that regulate various developmental processes, including seed development and germination, leaf expansion, stem elongation, and fruit patterning (Ogawa et al. 2003). Ethylene, a gaseous hydrocarbon hormone, regulates plant growth, senescence, and stress responses (Reid 1995). Auxin plays fundamental roles in regulating cell division, cell expansion, cell differentiation, lateral root formation, flowering, and tropic responses (Davies 1995; Seifertová et al. 2014). Jasmonic acid (JA) is a fatty acid (FA)‐derived phytohormone involved in plant defense and responses to various stresses (Ruan et al. 2019; J. Wang et al. 2020). Abscisic acid (ABA) regulates seed dormancy and guard cell function (Sano and Marion‐Poll 2021). Discovered in the 1960s and originally called dormin or abscissin (Wasilewska et al. 2008), ABA plays a crucial role in abiotic stress responses, including drought, osmotic, and cold stress (Einar Mantyla and Tapio Palva* 1995; Huang et al. 2018). While ABA is essential for stress adaptation, it also inhibits plant growth; high concentrations of exogenously applied ABA cause growth arrest, and endogenous ABA accumulation under stress conditions is often accompanied by growth reduction (Brookbank et al. 2021).

In this study, we investigated the role of phytohormones in regulating lipid metabolism in duckweed by conducting a screening using the aquatic plant *Lemna minor*, a widely used experimental model due to its rapid clonal propagation, simple body plan, and broad application in physiological, molecular, and biotechnological studies (Vulpe et al. 2025). Among the tested hormones, treatment with 1 µM ABA markedly increased TAG content in *L. minor*, accompanied by pronounced lipid class remodelling and coordinated transcriptional changes. These findings provide new insights into stress‐induced carbon allocation in duckweed and highlight the potential of hormonal regulation as a strategy for enhancing metabolite accumulation in aquatic plants.

## 2 Materials and Methods

### 2.1 Strains and Culture Conditions

*L. minor* BP1910791 was collected at the Tancheon freshwater fish wetland ecological garden (Seongnam‐si, Gyeonggi‐do, South Korea; 37°26'49.3''N, 127°07'06.0''E). *Spirodela polyrhiza* BP1910790 was obtained from Bibong Wetland Park in Hwaseong‐si, Gyeonggi‐do, South Korea (Kim et al. 2024). These strains are available from R&D Bio Resources at https://biorp.kribb.re.kr. *Lemna yungensis* was purchased from Daesang Aquarium (76, Jong‐ro 44‐gil, Jongno‐gu, Seoul, Republic of Korea). *Wolffia australiana* Wa8730 was kindly provided by Prof. Yang Jae Kang (Park et al. 2021). Duckweeds were cultured either in 1/2 strength Hoagland's (HG) liquid medium (Hoagland's No. 2 Basal Salt Mixture, H2395‐10L, Sigma) or 1/2 strength Schenk and Hildebrandt (SH) liquid medium (S0225.0050, Duchefa Biochemie) without sucrose at 23°C under a 16‐h light (130 μmol photons m^−2^ s^−1^)/8‐h dark photoperiod. *Arabidopsis thaliana* (Col‐0 ecotype) seeds were surface‐sterilised and germinated on 1/2 strength Murashige and Skoog (MS) agar plates containing 1% (w/v) sucrose, followed by cultivation at 23°C under a 16‐h light (130 μmol photons m^−2^ s^−1^)/8‐h dark photoperiod. For hormone treatment experiments, fronds were treated with 1‐aminocyclopropane‐1‐carboxylate (ACC; TCI; A1178), indole‐3‐acetic acid (IAA; Duchefa; I0901), methyl jasmonate (MeJA; Sigma; 392707), gibberellic acid (GA; Sigma; 48880), abscisic acid (ABA; Sigma; 90769), or *trans‐*zeatin (*t*Z; Sigma; Z0876). Stock solutions were prepared as follows: ACC was dissolved in distilled water; IAA, JA, GA, ABA, and tZ were dissolved in DMSO. All stock solutions were stored at −20°C and diluted to the final working concentration with growth medium immediately before use. For frond number quantification, daughter fronds that were similar in size to or larger than the mother fronds were counted as individual fronds, even if they remained physically attached.

### 2.2 Lipid Extraction and Quantification

Lipids were extracted and analysed as described previously (Sujeong Je et al. 2023; S. Je and Yamaoka 2022), with minor modifications. Briefly, duckweed samples were harvested and either freeze‐dried for 24 h using a freeze dryer (FDS, Ilshin Bio) or used as fresh tissue. Both freeze‐dried and fresh samples were ground into a fine powder using a Tissuelyser II (QIAGEN), and the resulting homogenates were subjected to total lipid extraction. To quantify TAGs, 1 mg of the total lipid extract was separated on a TLC plate using a solvent mixture of hexane:diethyl ether:acetic acid (80:30:1, v/v/v). To quantify membrane lipids, 1.5 mg of the total lipid extract was separated on a two‐dimensional TLC using a solvent mixture of acetone:toluene:methanol:water (80:30:20:10, v/v/v/v) and chloroform:methanol:acetic acid:water (170:25:25:4, v/v/v/v). Lipid spots were visualised under ultraviolet light after spraying with 0.01% (w/v) primuline (Sigma‐Aldrich) dissolved in acetone:water (4:1, v/v). TAG and individual membrane lipid bands were scraped from the plate, subjected to transesterification, and subsequently quantified by gas chromatography with flame ionisation detection (GC‐2030, Shimadzu, Korea) using an HP‐INNOWax capillary column (30 m × 0.25 mm i.d., 0.25 µm film thickness, Agilent Technologies).

### 2.3 *De novo* genome assembly and identification of homologues

Total genomic DNA was extracted using a SmartGene Plant DNA Extraction kit (SmartGene, Daejeon). Samples with a DNA integrity number >8.0 were used to construct sequencing libraries. Long‐read libraries were prepared using the SQK‐LSK110 kit (Oxford Nanopore Technologies) and sequenced on the ONT MinION platform. Short‐read libraries were prepared using the xGen™ DNA Library Prep EZ Kit (Integrated DNA Technologies) and sequenced on the Illumina NovaSeq. 6000 platform (Illumina). The long reads were subject to *de novo* genome assembly using NextDenovo v. 2.5.0 (Hu et al. 2024). The resulting contigs were polished using both ONT and Illumina reads with NextPolish v. 1.4.0 (Hu et al. 2020). The polished assembly was scaffolded with RagTag v. 2.1.0 (Alonge et al. 2019), using the *L. minor* 7210 genome as a reference (Ernst et al. 2025).

The statistics of the assembled genome were analysed using QUAST, and the completeness of the genome assembly was evaluated using Benchmarking Universal Single‐Copy Orthologs (BUSCO) v 5.2.2 with 3,236 single‐copy orthologs from the Liliopsida odb10 database (Manni et al. 2021; Mikheenko et al. 2018). Repetitive elements were annotated with RepeatMasker v. 4.1.5 (https://www.repeatmasker.org/RepeatMasker/) using a *de novo* repeat library generated by RepeatModeler v. 2.0.4 and the Repbase database (Rodriguez and Makałowski 2022). Protein‐coding gene prediction was carried out on the soft‐masked genome using the BRAKER 3 pipeline (Gabriel et al. 2023). To identify lipid metabolism‐related genes, predicted proteins were clustered into orthogroups alongside those from *A. thaliana, M. polymorpha*, and *O. sativa* using OrthoFinder v. 3.0.1 (Emms and Kelly 2019).

### 2.4 RNA Extraction and Quantification

Total RNA was extracted using a RNeasy Plant Mini Kit (QIAGEN) according to the manufacturer's instructions. Complementary DNA (cDNA) was synthesised from the extracted RNA using a reverse transcription kit (Thermo Fisher Scientific). Quantitative real‐time PCR (qRT‐PCR) was performed using the Step One Plus Real‐time PCR System (Applied Biosystems, Korea) with SYBR® Premix Ex Taq^TM^ (Takara, Japan) and the primer sets listed in (Table S1) to analyse lipid‐related and ABA‐responsive genes in *L. minor* (Tables S2, S3, S4). Gene expression levels were normalised to the ubiquitin C gene as an internal reference.

### 2.5 Starch Measurement

Duckweed fronds were harvested and freeze‐dried for 24 h. The dried tissues were ground into a fine powder using a TissueLyser II (QIAGEN). Starch content was measured using the Total Starch Assay Kit (Megazyme, K‐TSTA‐100A) according to the manufacturer's instructions. Briefly, soluble sugars were first removed by washing the powdered tissue with 80% (v/v) ethanol. The remaining pellet was hydrolysed enzymatically, and the released glucose was quantified colorimetrically at 570 nm using a microplate reader (TECAN). The starch concentration was calculated based on a standard curve prepared with known concentrations of glucose.

### 2.6 Visualisation of Lipid Droplets by Nile Red Staining and Confocal Microscopy

Lipid droplets were stained with 4 µg/mL Nile Red (Sigma‐Aldrich; from 0.1 mg/mL stock in acetone) and 0.02% (v/v) Tween‐20 (Sigma, P7949) in culture medium for 1 h. Duckweed fronds were cultured in liquid 1/2 HG medium containing 1 µM ABA dissolved in DMSO, or DMSO alone, for 3, 5 or 7 days. Fronds were then washed twice with culture medium. Nile Red‐stained fronds were visualised using a confocal microscope (LSM 900, ZEISS) under the following settings: excitation at 488 nm with 0.2% laser intensity, emission collection from 400 to 650 nm, and a master gain of 685 V.

### 2.7 Trypan Blue Staining

Duckweed fronds were cultured in liquid 1/2 HG medium containing 150 mM NaCl for 7 days, after a 3‐day pre‐culture in either 1/2 HG medium or 1/2 HG medium supplemented with 1 µM ABA dissolved in DMSO. After the treatment period, fronds were stained with 0.04% trypan blue (Sigma‐Aldrich; prepared from a 0.4% stock solution) in culture medium for 45 min and then destained in ethanol (Sigma‐Aldrich) for 1 day. Trypan blue‐stained duckweeds were observed using a stereomicroscope (M205 C, Leica).

## 3 Results

### 3.1 ABA Treatment Promotes Tag Accumulation in *L. minor*

**Figure 1:**
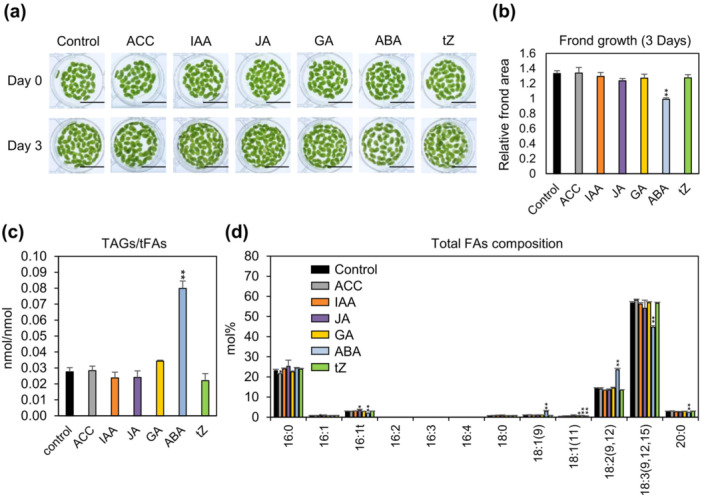
Effects of phytohormone treatments on growth and lipid metabolism in *L. minor*. (a) Representative images of *L. minor* fronds grown in 1/2 HG medium with 1 μM of each phytohormone: ACC, IAA, JA, GA, ABA, or tZ. Images were taken on Day 0 and Day 3. Scale bars represent 1 cm. (b) Relative increase in frond area over a 3‐day period. Error bars indicate the standard error (SE) from six biological replicates across three independent experiments. (c) Triacylglycerol (TAG) content normalised to total fatty acids (tFAs) in *L. minor* fronds after 3 days of treatment. For the control group, error bars show the SE from six replicates in three independent experiments. For the treatment groups, SE is based on four replicates in three independent experiments. (d) Total FA composition (mol%) in *L. minor* fronds following 3 days of treatment with the indicated phytohormones. Fronds were pre‐cultured in 1/2 HG medium for 7 days and then transferred to fresh medium containing the respective hormones. Error bars follow the same replication scheme as in (c). Statistical significance was assessed by two‐tailed *t*‐tests against the control group (*p < 0.1, **p < 0.05).

To investigate the regulatory potential of phytohormones in lipid metabolism, we first evaluated their effects on triacylglycerol (TAG) accumulation in *L. minor*. TAG is a major form of energy storage in plants and plays a crucial role in stress adaptation and development, making it a key indicator of metabolic shifts under phytohormone influence. Fronds of *L. minor* at an initial density of approximately 20 fronds per well were incubated in 1/2 HG medium containing ABA, ACC, IAA, JA, GA, or tZ for 3 days to evaluate the effects of phytohormones on TAG accumulation. The 1/2 HG medium was chosen over 1/2 SH for these experiments because it minimised the formation of white fronds, which could otherwise affect TAG content (Figure S1). Phenotypic analysis revealed that *L. minor* fronds treated with 1 μM ABA for 3 days did not show a noticeable increase in frond area compared with those treated with other phytohormones or the control (Figure 1a,b), consistent with findings in *A. thaliana*, rice, and tomato, where basal ABA levels have been shown to inhibit growth and cell proliferation (Brookbank et al. 2021). Among the treatments, ABA resulted in the highest accumulation of the storage lipid TAG, with the TAG‐to‐total fatty acid (tFA) ratio increasing 2.9‐fold compared with the control (Figure 1c). In contrast, treatments with other phytohormones had no significant effect on growth, and caused only minor changes in the TAG‐to‐tFA ratio. To further assess ABA‐induced changes in lipid metabolism, we analysed the tFA pool, representing all fatty acids in the cell regardless of lipid class, including those esterified in storage and membrane lipids as well as any free fatty acids. ABA treatment altered the composition of tFAs, notably decreasing the level of 18:3 while increasing the levels of 18:1 and 18:2 (Figure 1d). Given the substantial increase in TAG upon ABA treatment, we next investigated the mechanisms underlying this response. To assess potential solvent effects, DMSO‐treated mock controls were included (Figure S2). DMSO alone did not affect frond growth, fatty acid composition, or TAG accumulation. Unless otherwise stated, control conditions refer to untreated fronds without DMSO.

To investigate whether higher concentrations of ABA could further enhance TAG accumulation, lipid analysis was conducted in 1/2 HG medium supplemented with 1 μM, 5 μM, or 10 μM ABA. At concentrations above 5 μM, *L. minor* fronds developed white spots (Figure S3a), indicating that excessive ABA may negatively affect plant health. While all ABA treatments increased TAG accumulation compared with the control, TAG levels were only slightly higher at 5 and 10 μM than at 1 μM (Figure S3b). Additionally, the total tFA composition remained unchanged across all ABA concentrations tested (Figure S3c). Based on these findings, 1 μM ABA was selected for subsequent experiments to balance effective TAG induction with minimal physiological stress.

### 3.2 Prolonged ABA Treatment Enhances TAG Accumulation

**Figure 2:**
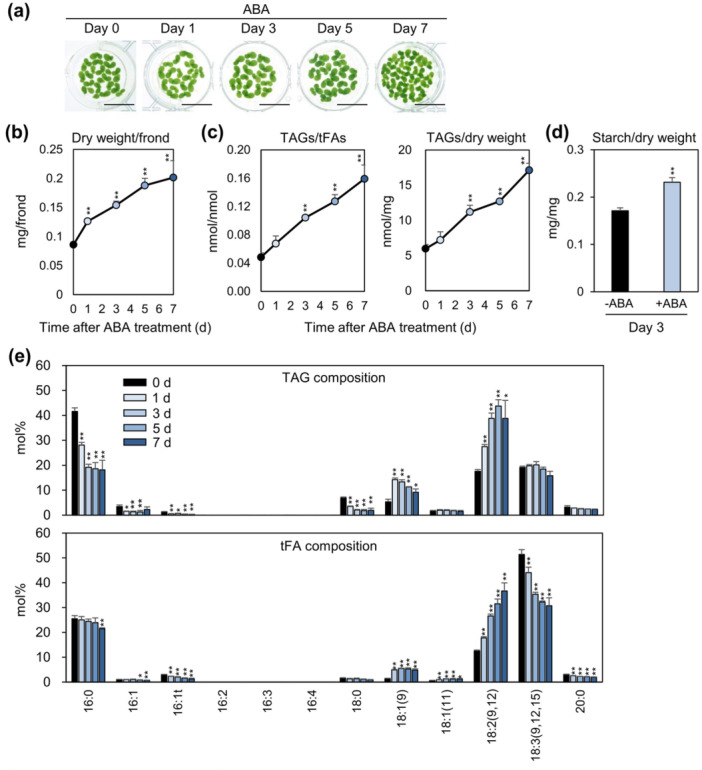
Time course analysis of lipid composition in *L. minor* under ABA treatment. (a) Representative images of fronds cultivated in 1/2 HG medium containing 1 μM ABA for 0, 1, 3, 5, or 7 days. Scale bars indicate 1 cm. (b) Dry weight of fronds treated with 1 μM ABA, normalised by the number of stage 7 fronds. Error bars show standard error (SE) calculated from six replicates in two independent experiments. (c) Triacylglycerol (TAG) content of fronds treated with 1 μM ABA, normalised by either total fatty acids (tFAs) or dry weight. Error bars indicate SE based on four replicates in two independent experiments. (d) Starch content in fronds treated with 1 μM ABA for 3 days, normalised by dry weight. Error bars represent SE from nine replicates in two independent experiments. (e) FA composition of TAGs and tFAs in fronds treated with 1 μM ABA for 0, 1, 3, 5, or 7 days. Fronds were pre‐cultured in 1/2 HG medium for 7 days before ABA treatment. Lipids were extracted and analysed at each time point. Error bars indicate SE based on four replicates in two independent experiments. Statistical significance was assessed using a two‐tailed *t*‐test compared to the 0‐day control group (*p < 0.1, **p < 0.05). [Color figure can be viewed at wileyonlinelibrary.com]

To determine whether prolonged ABA treatment increases TAG accumulation, we examined its effect over multiple time points in *L. minor*. Figure 2a shows the time course of frond growth under ABA treatment. Visible frond expansion was suppressed during the early phase of treatment (up to Day 3), consistent with the phenotypic analysis shown in Figure 1. Notably, the growth‐inhibitory effect of ABA gradually weakened after Day 5 and was minimal by Day 7, when an increase in frond area became apparent. To enable accurate comparison of growth responses, we analysed fronds that had reached full size within the pouch but had not yet emerged (Figure S4). These fronds were illuminated from below to visualise internal structures clearly, allowing consistent staging across samples. We confirmed the significant growth suppression in daughter frond development compared with untreated controls, with daughter frond emergence particularly inhibited (Figure S4), suggesting a stage‐dependent inhibitory effect of ABA on growth, most evident in early daughter fronds.

In contrast, Figure 2b shows that dry weight increased from Day 1 onward following ABA treatment, despite the suppression of visible frond expansion during the early phase. This indicates that ABA promotes biomass accumulation even when apparent growth is inhibited. Consistent with this increase in biomass, TAG levels also began to increase from Day 1 and continued to accumulate through Day 7 (Figure 2c). In addition, total starch content was significantly elevated by ABA treatment at Day 3 (Figure 2d), suggesting that the increase in dry weight was largely due to the accumulation of both storage molecules, TAGs and starch. Analysis of tFA composition revealed a significant reduction in 18:3 over the 7‐day ABA treatment period (Figure 2e, lower panel). In contrast, within the TAG fraction, 18:3 levels were maintained, and 18:2 content steadily increased throughout the treatment (Figure 2e, upper panel). Moreover, the proportion of 16:0 in TAGs was markedly reduced. These results suggest that TAG production under ABA treatment preferentially utilises 18:2 and 18:3 as major FA substrates in *L. minor*, indicating the lipid sources contributing to TAG formation.

### 3.3 ABA Enhances FA Synthesis and Redirects Chloroplast Lipid Pools Toward TAG Biosynthesis in *L. minor*

**Figure 3:**
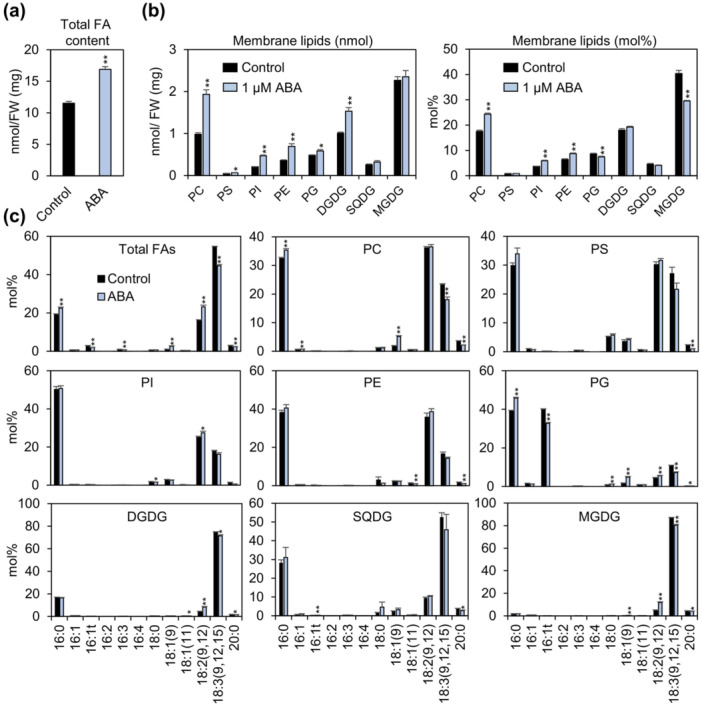
Effects of ABA treatment on total fatty acid (tFAs) and membrane lipid profiles in *L. minor*. (a) Total FA content normalised to fresh weight (FW) in *L. minor* fronds treated with 0 μM (control) or 1 μM ABA for 3 days. (b) Quantification of membrane lipid species is shown as nmol per milligram of FW (left) and as mol percent of total membrane lipids (right) after treatment with 0 μM or 1 μM ABA. (c) FA composition profiles of tFAs and individual membrane lipid classes, including PC, PS, PI, PE, PG, DGDG, SQDG, and MGDG, in *L. minor* fronds after 3 days of ABA treatment. Membrane lipids were extracted and analysed by gas chromatography with flame ionisation detection (GC‐FID). Error bars indicate standard error (SE) based on three replicates from one independent experiment. Statistical significance was assessed by a two‐tailed *t*‐test (*p < 0.1, **p < 0.05). DGDG, digalactosyldiacylglycerol; MGDG, monogalactosyldiacylglycerol; PC, phosphatidylcholine; PS, phosphatidylserine; PI, phosphatidylinositol; PE, phosphatidylethanolamine; PG, phosphatidylglycerol; SQDG, sulfoquinovosyldiacylglycerol. [Color figure can be viewed at wileyonlinelibrary.com]

Environmental stresses are known to significantly affect membrane lipid composition in plants and algae, often leading to remodelling of both plasma and organelle membranes (Moellering et al. 2010; Welti et al. 2002; Yamaoka et al. 2019). Given that ABA altered both tFA composition and TAG content in *L. minor*, we hypothesised that ABA treatment would also impact membrane lipid profiles. To test this, membrane lipids were extracted and analysed from *L. minor* fronds treated with 1 μM ABA for 3 days and compared with untreated control. Total FA content was significantly increased following ABA treatment (Figure 3a), suggesting an overall increase in membrane lipid levels. Indeed, the absolute amounts (nmol) of most membrane lipids, including phospholipids and galactolipids, were elevated by ABA treatment (Figure 3b, left). Evaluating lipid composition in molar percentage (mol%) enables detection of relative shifts among lipid species, which may reflect membrane remodelling or metabolic reallocation, independent of total lipid quantity. The molar percentage of monogalactosyldiacylglycerol (MGDG), a major chloroplast lipid enriched in 18:3 and 18:2 (Figure 3c), was markedly reduced to approximately 73% of the control, making it the most prominently decreased lipid class (Figure 3b, right). This reduction coincides with the accumulation of 18:3 and 18:2 in TAGs under ABA treatment (Figure 2e), suggesting that MGDG remodelling may contribute polyunsaturated FAs for TAG biosynthesis. In addition, the molar percentage of phosphatidylcholine (PC), a key extraplastidial membrane lipid, was increased to approximately 139% of the control following ABA treatment, and its absolute amount (nmol) also showed a substantial increasing trend (Figure 3b). This suggests that PC may act as a site for FA remodelling and serve as an intermediate in the metabolic flux toward TAG synthesis. These findings indicate that both MGDG and PC contribute to TAG accumulation under ABA‐induced conditions by redirecting membrane lipid pools and facilitating FA flux toward storage lipids.

### 3.4 ABA Treatment Induces TAG Accumulation in Mother Fronds and Chloroplast‐Containing Roots

**Figure 4:**
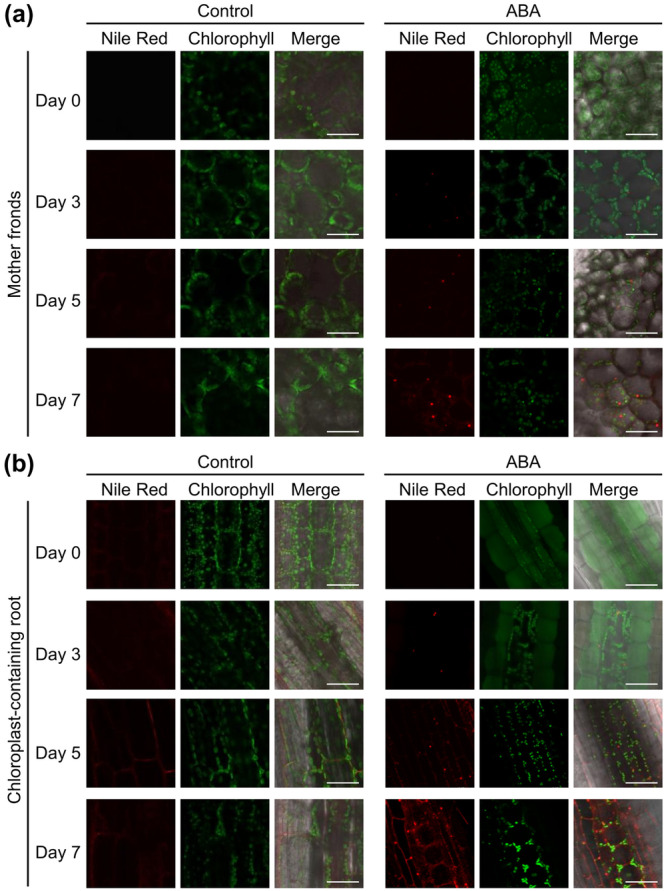
Visualisation of lipid droplets in *L. minor* during ABA treatment. Representative confocal microscopy images of the abaxial surface of (a) mother fronds and (b) chloroplast‐containing root tissues under control conditions (left) or following ABA treatment (right). Lipid droplets were stained with Nile Red (red), and chlorophyll autofluorescence is shown in green. Merged images display the overlap of both signals. Scale bars = 50 µm. [Color figure can be viewed at wileyonlinelibrary.com]

To identify the specific tissues where lipid accumulation occurs, *L. minor* fronds containing daughter fronds that had reached half their size and remained partially enclosed in the mother pouch, corresponding to stage 5 in *S. polyrhiza* in Kim et al. (2024), were treated with 1 μM ABA and stained with Nile Red, a fluorescent dye that labels nonpolar lipids. In control samples, lipid droplets were not detected in either mother fronds or chloroplast‐containing roots throughout the observation period (Figure 4). Lipid droplets were observed in the mother fronds starting from Day 3 after ABA treatment, with both their size and number increasing by Day 7 after ABA treatment (Figure 4). Many of these lipid droplets appeared to be located adjacent to or in close proximity to chloroplasts in mother fronds. In contrast, no lipid droplet was detected in daughter fronds at this developmental stage throughout the observation period.

Interestingly, lipid droplets were also observed in chloroplast‐containing roots, with the highest number recorded on Day 7 after ABA treatment, similar to the pattern observed in the mother fronds (Figure 4). Notably, at Day 7, numerous small lipid droplets were present in both the mother fronds and chloroplast‐containing roots. The observed temporal pattern of lipid droplet formation corresponded well with the biochemical quantification of TAG accumulation (Figure 2c). These observations suggest that ABA induces lipid droplet formation preferentially in chloroplast‐containing source tissues, such as mother fronds and roots, but not in sink tissues such as daughter fronds, highlighting a potential link between chloroplast presence and lipid storage under stress conditions.

### 3.5 ABA Treatment Induces Expression Changes in *DGAT* and *FAD* Genes in *L. minor*

To investigate whether exogenous ABA treatment modulates the expression of genes involved in lipid metabolism in *L. minor*, we first generated a draft genome assembly to enable gene identification. Integration of 41 Gb of long‐read ONT data with an N50 of 14.2 kb and 18 Gb of Illumina short‐read data yielded an initial genome assembly with a genome size of 762.2 Mb, comprising 799 contigs. Scaffolding with the *L. minor* 7210 reference genome produced 21 pseudo‐chromosomes, representing a scaffolded genome size of 480 Mb with an N50 of 24.4 Mb. Genome completeness was assessed using the BUSCO approach with the Liliopsida odb10 dataset. BUSCO analysis revealed that 78.8% of the core genes were completely captured in the assembled genome (Table S5), a level of genome completeness comparable to the 72.5% reported for the *L. minuta* genome (Abramson et al. 2022). Using the BRAKER3 pipeline, we annotated 23,028 protein‐coding genes in the *L. minor* genome.

**Figure 5:**
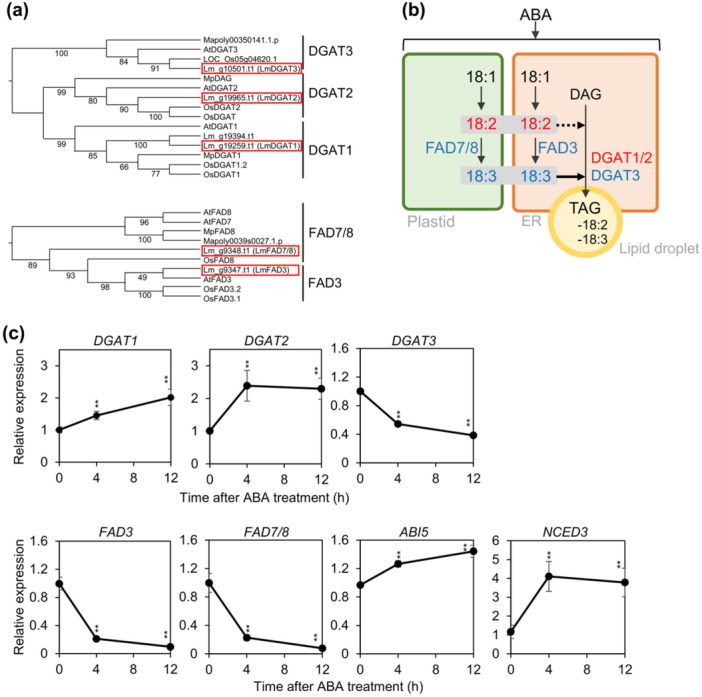
Expression levels of lipid‐related and ABA‐responsive genes in *L. minor* following ABA treatment. (a) Phylogenetic tree showing the relationships among diacylglycerol acyltransferases (DGATs) and fatty acid desaturases (FADs). Bootstrap support values are indicated at the branch points. (b) Proposed model of triacylglycerol (TAG) accumulation upon ABA treatment. The pathway highlights desaturation steps occurring in the plastid and endoplasmic reticulum (ER), and subsequent TAG formation in lipid droplets (LDs). (c) Relative expression levels of acyltransferase genes (*DGAT1, DGAT2* and *DGAT3*), desaturase genes (*FAD3* and *FAD7/8*), and ABA‐responsive genes (*ABI5* and *NCED3*) in *L. minor* treated with 1 μM ABA for the indicated times. Gene expression was normalised to UBC expression. Error bars indicate standard error (SE). For *DGAT1* at 0 and 12 h, SE was calculated from eleven biological replicates; for *DGAT1* at 4 h, from ten replicates; for *DGAT3*, from six replicates; and for *NCED3*, from eleven replicates. For all other genes and time points, SE was calculated from five replicates. Statistical significance was assessed using a two‐tailed *t*‐test (*p < 0.1, **p < 0.05). ABI, abscisic acid insensitive; DGAT, diacylglycerol acyltransferase; FAD, fatty acid desaturase; NCED, 9‐cis‐epoxycarotenoid dioxygenase; UBC, ubiquitin C. [Color figure can be viewed at wileyonlinelibrary.com]

Transcript levels were then analysed using this genome resource. Since ABA treatment led to TAG accumulation and reduced 18:3 levels (Figures 1 and 2), we focused on genes encoding diacylglycerol acyltransferases (DGATs) and FA desaturases (FADs), which may contribute to these phenotypes. Homologues of *A. thaliana DGAT* and *FAD* genes were identified in *L. minor* through comparative genomic and phylogenetic analyses using the protein sequences from *L. minor*, *A. thaliana, M. polymorpha*, and *O. sativa*. We identified two *AtDGAT1* homologues (Lm_g19259.t1 and Lm_g19394.t1), one *AtDGAT2* homologue (Lm_g19965.t1), one AtDGAT3 homologue (Lm_g10501.t1), one *AtFAD3* homologue (Lm_g9347.t1), and one *AtFAD7/8* homologue (Lm_g9348.t1) in the *L. minor* genome (Figure 5a,b). Among the *AtDGAT1* homologues, Lm_g19259.t1 showed the highest sequence similarity to *AtDGAT1* and was selected for further analysis.

Following ABA treatment, the expression of both *LmDGAT1* and *LmDGAT2* was significantly upregulated (Figure 5c), suggesting that ABA enhances TAG production through transcriptional activation of *DGAT* genes. In contrast, expression of *LmDGAT3* was significantly downregulated at 4 and 12 h after ABA treatment (Figure 5c), indicating that DGAT3 is unlikely to contribute positively to ABA‐induced TAG accumulation in *L. minor*. In addition, the expression of the fatty acid desaturase genes *LmFAD3* and *LmFAD7/8* were significantly downregulated at 4 and 12 h after ABA treatment (Figure 5c), indicating that ABA suppresses desaturase expression, thereby reduces 18:3 FA synthesis. To confirm activation of the ABA signalling pathway, we examined the expression of ABA‐responsive genes *ABI5* and *NCED3* (Finkelstein and Lynch 2000; Thompson et al. 2000). Both genes were upregulated following ABA treatment (Figure 5c), confirming effective induction of ABA signalling.

### 3.6 Lipid Droplet Accumulation Following ABA Treatment and Its Possible Role in Salt Stress Response

To clarify the physiological role of ABA‐induced phenotypes on the subsequent growth of *L. minor*, we first monitored the dynamics of TAGs stored in lipid droplets following ABA exposure. Fronds were treated with ABA for 3 days, then transferred to 1/2 HG medium without ABA and cultured for an additional 7 days (Figure S5a). TAG levels, which had accumulated during ABA treatment, returned to control levels after ABA was withdrawn (Figure S5b), suggesting that lipid droplets were remobilized to support cellular lipid metabolism during recovery. To assess whether ABA pretreatment could enhance tolerance to later stress conditions, fronds cultured for 3 days in either 1/2 HG medium with or without ABA were transferred to medium containing 150 mM NaCl and cultured for 7 days. Trypan blue staining revealed fewer dead cells in the group pretreated with ABA (Figure S5c). Taken together, ABA treatment primes *L. minor* for salt stress by inducing lipid droplet accumulation, which can be remobilized to support recovery following stress relief. In parallel, ABA pretreatment enhances salt stress tolerance, indicating that ABA primes *L. minor* for future environmental challenges by building up an energy reservoir in lipid droplets.

### 3.7 Salt Stress Induces TAG Accumulation

ABA levels are known to increase in response to various stress conditions, including salt stress (Golldack et al. 2014). To examine whether TAG accumulation observed under ABA treatment also occurs under physiologically relevant conditions, fronds were treated with 50, 100 and 150 mM NaCl (Figure S6a). Although NaCl treatment inhibited frond proliferation, it increased dry weight per frond (Figure S6b), resembling the effects of ABA treatment (Figure 2b). Consistently, TAG levels rose with increasing NaCl concentrations (Figure S6c). FA composition analysis revealed similar trends to those under ABA treatment (Figure S6d), including an increase in 18:2 and a decrease in 18:3 in tFA, as well as an increase in 18:2 and a partial reduction in 16:0 in TAGs. These results suggest that NaCl induces lipid remodelling in *L. minor* in a manner similar to ABA treatment. Given that salt stress activates ABA signalling, the observed lipid changes are likely mediated, at least in part, through ABA‐dependent pathways.

### 3.8 *L. minor* Exhibits Uniquely Strong TAG Accumulation in Response to ABA Among Duckweeds and *A. thaliana*

**Figure 6:**
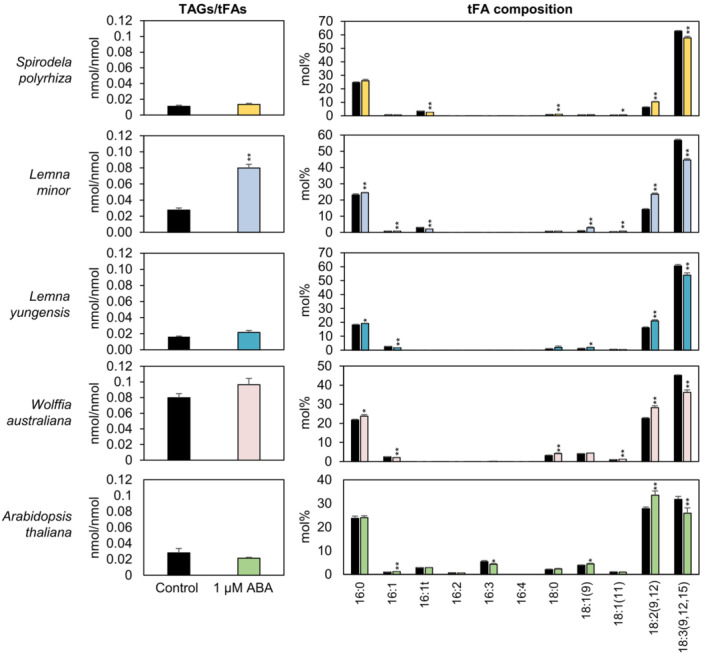
TAG‐to‐total fatty acid (tFA) ratios and FA composition in response to ABA treatment across duckweed species and *A. thaliana*. TAG‐to‐tFA ratios and tFA compositions were analysed after 3 days of treatment with 1 μM ABA in the following duckweed species: *S. polyrhiza*: Error bars for the control represent standard error (SE) from five biological replicates across three independent experiments; ABA‐treated samples from four biological replicates across three experiments. *L. minor*: Error bars represent SE from six biological replicates across three experiments. *L. yungensis*: Error bars represent SE from five biological replicates from one experiment. *W. australiana*: Error bars for the control and ABA‐treated samples represent SE from five and eight biological replicates, respectively, across two independent experiments. *A. thaliana*: Seedlings were pre‐grown for 7 days in 1/2 MS medium before ABA treatment. Each biological replicate included 15 seedlings. Error bars represent SE from three biological replicates across two independent experiments. Statistical significance was determined using a two‐tailed *t*‐test (*p < 0.1, **p < 0.05). [Color figure can be viewed at wileyonlinelibrary.com]

To determine whether ABA‐induced TAG accumulation is a conserved physiological response among duckweeds and land plants, we treated four duckweed species, including *Spirodela polyrhiza, L. minor, Lemna yungensis, and Wolffia australiana*, as well as the model land plant *A. thaliana* with ABA and analysed their lipid profiles. All species showed a similar shift in FA composition, with increased 18:2 and decreased 18:3 (Figure 6). However, a pronounced and robust increase in TAG levels was detected exclusively in *L. minor*. In contrast, *S. polyrhiza*, *L. yungensis*, and *W. australiana* showed no significant TAG accumulation, despite undergoing FA remodelling. Previous studies using higher ABA concentrations (e.g., 10 μM) have suggested that TAG accumulation may occur in *A. thaliana* (Kong et al. 2013; Lee et al. 2019), but under our experimental conditions with 1 μM ABA, *A. thaliana* did not exhibit a statistically significant increase in TAG levels (Figure 6). These findings demonstrate that *L. minor* possesses an exceptionally high sensitivity to ABA‐mediated TAG biosynthesis, highlighting species‐specific differences in lipid metabolic responses to ABA among duckweeds and between aquatic and terrestrial plants.

## 4 Discussion

In this study, we investigated the effects of plant hormones on lipid remodelling in duckweeds, with a particular focus on TAG accumulation, FA composition, and membrane lipid profiles. Through physiological, biochemical, and transcript analyses, *L. minor* was identified as the species exhibiting the most prominent response to ABA, characterised by substantial TAG accumulation. This conclusion is supported by the following key findings: (1) Among the phytohormones tested, ABA treatment induced the most pronounced lipid metabolic response in *L. minor*, including a 2.9‐fold increase in the TAG‐to‐tFA ratio and a marked reduction in 18:3 content at Day 3 (Figure 1), with these effects persisting through Day 7 (Figure 2). These changes were accompanied by increased dry weight, likely due to enhanced accumulation of both lipids and starch (Figure 2). (2) Transcript analysis revealed that ABA treatment significantly upregulated the expression of *DGAT1* and *DGAT2*, key genes involved in TAG biosynthesis, while downregulating *FAD3* and *FAD7/8*, which encode desaturases responsible for 18:3 production (Figure 5) (Carro et al. 2022). (3) Membrane lipid profiling showed a substantial reduction in MGDG, a major chloroplast lipid enriched in 18:3, and a concomitant increase in PC, an extraplastidial lipid (Figure 3). These shifts suggest a redirection of chloroplast‐derived lipid pools toward TAG biosynthesis. Consistent with this, lipid droplets were prominently observed adjacent to chloroplasts in chloroplast‐containing tissues such as mother fronds and roots (Figure 4). (4) Finally, cross‐species comparisons further revealed that substantial TAG accumulation in response to 1 μM ABA was largely restricted to *L. minor*, whereas other duckweed species (*S. polyrhiza, L. yungensis*, and *W. australiana*) and the model land plant *A. thaliana* showed minimal or no response under the same conditions (Figure 6). These results suggest that *L. minor* exhibits species‐specific responsiveness to ABA‐mediated lipid remodelling under the tested conditions. Collectively, our findings highlight *L. minor* as a promising model for investigating ABA‐regulated lipid metabolism in aquatic plants. The species‐specific nature of this response may offer insights into the evolutionary and ecological significance of lipid remodelling under stress and inform strategies for enhancing stress tolerance or lipid production in plant biotechnology.

### 4.1 Chloroplast MGDG May Contribute to TAG Biosynthesis Under ABA Treatment, Possibly Through Recycling or Redirection of FAs

ABA treatment led to a significant increase in TAG accumulation in *L. minor*, accompanied by elevated tFA levels and increased amounts of most membrane lipids, except for a marked reduction in MGDG (Figures 2c and 3b). FA composition analysis showed that TAGs were predominantly enriched in 18:2 and 18:3, with 18:3 being the major acyl chain in MGDG (Figures 2e and 3c). These observations suggest that MGDG is mobilised as a source of FAs for TAG biosynthesis under ABA‐induced stress. This mechanism is consistent with stress responses in other photosynthetic organisms. In *A. thaliana*, the chloroplast‐localised lipase HEAT INDUCIBLE LIPASE1 (HIL1) is activated under heat stress and releases 18:3 FA from MGDG, contributing to TAG accumulation (Higashi et al. 2018). Under freezing stress, the SENSITIVE TO FREEZING 2 (SFR2) enzyme converts MGDG into diacylglycerol (DAG) and oligogalactolipids, providing substrates for TAG synthesis (Moellering et al. 2010). In microalgae such as *Chlamydomonas reinhardtii*, the plastidial lipase PGD1 hydrolyzes MGDG and is essential for TAG accumulation during nitrogen deprivation stress (Li et al. 2012). Loss of *PGD1* impairs galactoglycerolipid turnover, reduces TAG production, and increases ROS, highlighting a critical role for MGDG‐derived FAs in stress adaptation (Du et al. 2018; Li et al. 2012). These findings point to a conserved strategy across aquatic algae and terrestrial plants, wherein stress‐induced mobilisation of chloroplast MGDG supports TAG biosynthesis. In *L. minor*, ABA‐induced lipid remodelling likely employs a similar mechanism.

In addition to the breakdown of pre‐existing MGDG and redirection of its FAs toward TAG synthesis, it is also important to consider that newly synthesised plastidial FAs, originally destined for MGDG assembly, may instead be rerouted toward TAG production. *L. minor* is classified as an 18:3‐type plant, similar to rice, in which a portion of chloroplast‐synthesised FAs is exported to the endoplasmic reticulum (ER), where they are incorporated into phosphatidic acid (PA) and diacylglycerol (DAG), precursors for various membrane lipids (Cook et al. 2021). Under normal conditions, some of these ER‐derived DAG molecules are typically reimported into the chloroplast for MGDG synthesis via MGD enzymes. However, ABA treatment may suppress this reimport, thereby retaining more DAG in the ER and redirecting it toward PC and TAG biosynthesis rather than chloroplast lipid assembly. The observed increase in PC, the major extraplastidial membrane lipid, is notable in this context. PC not only serves as a structural lipid but also serves as a key site for FA modification and exchange. Through lysophosphatidylcholine acyltransferase (LPCAT)‐mediated acyl editing, PC becomes enriched with plastid‐derived polyunsaturated FAs, and PC‐derived DAG can be acylated by DGAT1, DGAT2, or phospholipid:diacylglycerol acyltransferase (PDAT) to produce TAGs (Bates and Shockey 2025). Thus, ABA‐induced PC accumulation in *L. minor* may reflect its role as a metabolic hub that channels newly synthesised plastidial FAs into storage lipid formation.

Given that the absolute content of MGDG remained unchanged under ABA treatment (Figure 3b left), the second route, in which *de novo*‐synthesised plastidial FAs are rerouted toward PC and TAG formation via ER‐localised lipid pathways, may appear to be the dominant contributor. However, the unchanged net MGDG level does not preclude active turnover, suggesting that a recycling route could also be activated. Such remodelling would facilitate efficient storage lipid formation under stress conditions and underscores the metabolic flexibility and adaptive capacity of lipid metabolism in *L. minor*.

### 4.2 Species‐Specific Lipid Accumulation in *L. minor* Under ABA‐Induced Growth Inhibition

ABA has traditionally been recognised as a growth‐inhibitory hormone; for instance, it suppresses germination and overall plant development in *A. thaliana* (Bi et al. 2020; Brookbank et al. 2021). In our study, ABA similarly inhibited growth in *L. minor*, particularly in daughter fronds at early stages (Figure S4). Although frond expansion was suppressed, dry weight increased during prolonged exposure (Figure 2b), suggesting that ABA can promote the accumulation of biomass components.

ABA is known to regulate starch biosynthesis in monocot species, such as rice, where it enhances grain filling and seed quality by modulating carbohydrate metabolism (J.‐D. Wang et al. 2024; Z. Wang et al. 2015). Notably, ABA‐induced TAG accumulation has previously been documented only in *A. thaliana*, where it promotes a modest increase in TAG levels under stress conditions. For example, 10 µM ABA treatment induced TAG accumulation, visualised by thin‐layer chromatography (TLC), although precise lipid measurements were not provided (Kong et al. 2013). A subsequent study by Lee et al. (2019), using 10 µM ABA, reported a modest increase ( ~ 16.7%) in TAG content, quantified more precisely by gas chromatography. While these studies support a role for ABA in stimulating TAG biosynthesis, the concentrations used are above the physiological range of endogenous ABA (typically 0.1–2 µM under stress; (Jaleh Daie 1981; Luo et al. 1992). Although such concentrations can be useful for probing hormonal effects, further investigation is needed to understand how plants respond to more physiologically relevant ABA levels.

In line with these reports, our results demonstrate that 1 µM ABA treatment in *L. minor* leads to the accumulation of both starch (1.4‐fold; Figure 3d) and TAGs (2.9‐fold; Figure 1c), suggesting a broader role for ABA in carbon allocation toward storage reserves in this species. Interestingly, this ABA‐induced TAG accumulation was observed predominantly in *L. minor*. The other species we tested, *S. polyrhiza, L. yungensis*, *W. australiana*, and *A. thaliana*, did not show comparable increases in TAG content under the same ABA concentration (Figure 6).

Two hypotheses may explain the species‐specific lipid remodelling observed under ABA treatment. First, all species tested likely perceive ABA, but the transcriptional or metabolic pathways activated downstream differ, enabling *L. minor* to channel more carbon into TAG biosynthesis. For example, although *L. yungensis* belongs to the same genus as *L. minor*, it did not exhibit any increase in TAG content under the same conditions (Figure 6), indicating that *L. yungensis* may respond in a manner distinct from TAG production under ABA treatment. In *Landoltia punctata*, spraying 0.5 mM ABA (estimated to yield ~1.78 µM in the medium) induced a 2.6‐fold increase in starch content (Liu et al. 2018). Such variation in downstream responses could also occur within *L. minor* ecotypes. For instance, *L. minor* reported by McLaren and Smith (1976) accumulated over 500% more starch after 7 days of 1 µM ABA treatment, markedly higher than the starch accumulation observed in our study at Day 3. Although these values are not directly comparable due to differences in experimental duration, they point to substantial interspecific and intraspecific variability in the metabolic routing of carbon under ABA signalling. Second, the observed specificity may be due to differences in the threshold ABA concentration required to trigger a metabolic response. While 1 μM ABA was sufficient to induce growth inhibition in *L. minor* (Figures 1 and S3), it was insufficient to elicit similar responses in *S. polyrhiza* (Figure S7). *L. gibba*, another species within the *Lemna* genus, responds to as little as 0.5 μM ABA with a 50% reduction in relative growth rate, indicating a stronger inhibitory effect than observed in our study (Thorsteinsson and Eliasson 1990). These findings suggest that both variation in downstream metabolic pathways and differences in ABA sensitivity contribute to the diversity of ABA‐mediated lipid metabolic responses among duckweeds. Elucidating the underlying mechanisms will be essential to understanding how ABA signalling shapes carbon allocation across species.

### 4.3 Transcript‐Level Regulation Underlying ABA‐Induced TAG Biosynthesis and FA Remodelling in *L. minor*

Our data indicate that the enhanced TAG accumulation under ABA treatment is associated with the upregulation of key TAG biosynthetic genes. DGATs catalyse the final and committed step of TAG biosynthesis, transferring an acyl group from acyl‐CoA to DAG to form TAG (Chapman and Ohlrogge 2012; Lardizabal et al. 2001). In our study, both *DGAT1* and *DGAT2* were significantly upregulated following ABA treatment (Figure 5), supporting the role of ABA in promoting TAG synthesis and its deposition in lipid droplets, in accordance with known plant lipid metabolic pathways (Torabi et al. 2021). While ABA treatment activated ER‐associated TAG biosynthesis through the upregulation of *DGAT1*, *DGAT2* and *DGAT3* expression was reduced, suggesting that cytosolic/chloroplastic *DGAT3* participates in a distinct TAG synthesis route that is less required under ABA‐induced ER‐centric lipid storage, and is therefore downregulated, possibly through feedback from increased TAG levels and altered acyl‐CoA fluxes (Carro et al. 2022). Lipid droplets are multifunctional organelles primarily involved in the storage of neutral lipids for energy and carbon reserves. Lipid droplet accumulation in seeds supports germination (Y. Yang and Benning 2018), and in vegetative tissues, it can occur in response to abiotic stresses such as drought, oxidative stress, and osmotic imbalance (Bouchnak et al. 2023). Building on these stress‐induced cases, our study shows that the phytohormone ABA, a major regulator of stress responses, can directly trigger lipid droplet formation in *L. minor* (Figure 4). In this work, we not only quantified ABA‐induced lipid droplet accumulation (Figures 1 and 2) but also characterised membrane lipid reprogramming (Figure 3).

ABA treatment also altered FA composition, most notably reducing the proportion of 18:3 in the tFA pool (Figures 1d and 2e). This decrease can be attributed to the downregulation of *FAD3* in ER and *FAD7/8* in chloroplast, which mediate the desaturation of 18:2 to 18:3 (Figure 5). Interestingly, while 18:3 levels declined in tFAs, their levels remained relatively stable within the TAG fraction. This may be due to the substrate specificity of certain DGAT isoforms, which preferentially incorporate polyunsaturated FAs such as 18:3 into TAGs. For example, GmDGAT1A from soybean has been shown to preferentially use 18:3‐acyl CoA for TAG synthesis (B. Chen et al. 2016). This observation suggests that TAG accumulation is not only a consequence of FA availability but also of selective enzymatic processing, likely serving to sequester PUFAs within lipid droplets to protect them from reactive oxygen species under stress conditions.

How can ABA‐mediated TAG accumulation occur? Two previous studies have implicated ABI transcription factors in the regulation of lipid biosynthetic genes. ABI4 and ABI5 synergistically activate DGAT1 expression under stress, promoting TAG accumulation in Arabidopsis seedlings (Kong et al. 2013). ABI3 promotes TAG accumulation independently of FUS3, likely by strongly upregulating lipid droplet protein genes and WRINKLED1 (Z. Yang et al. 2022). Although neither study reported ABA‐driven TAG accumulation in wild‐type plants, their findings strongly support our observation of this phenomenon in *L. minor* under ABA treatment. Furthermore, in our study, the observed induction of ABI5 expression suggests a regulatory role for the ABA signalling pathway in linking stress responses with lipid metabolism.

In addition to neutral lipid metabolism, ABA treatment also modulated membrane lipid composition (Figure 3). In *L. minor*, ABA markedly increased the absolute amounts (nmol) of PC, phosphatidylinositol (PI), phosphatidylethanolamine (PE), and digalactosyldiacylglycerol (DGDG) (Figure 3b left). This pattern is largely consistent with previous findings in *Physcomitrium patens*, where ABA enhanced PC, PI and DGDG accumulation to support desiccation tolerance (Yu et al. 2024). Together, these results suggest that ABA can trigger partial membrane lipid remodelling in *L. minor*, potentially contributing to stress resilience, although the magnitude and functional significance may differ from terrestrial species depending on hydration status and ecological niche.

### 4.4 Stress‐Induced TAG Accumulation and Its Reversibility in *L. minor*

In *L. minor*, ABA‐induced TAG accumulation was fully reversible (Figure S5b). When fronds were transferred from ABA‐containing to ABA‐free medium, TAG levels quickly returned to those of untreated controls, indicating that sustained TAG biosynthesis requires continuous ABA signalling and is an active, regulated process rather than passive accumulation. This dynamic regulation reflects the broader role of ABA as a central stress hormone mediating plant responses to environmental cues such as drought, salinity, and osmotic stress (Rai et al. 2024). Because these environmental stresses often elevate endogenous ABA levels, they can elicit similar metabolic adjustments, including TAG biosynthesis. In higher plants, drought and salinity commonly induce TAG biosynthesis as part of stress adaptation (Liang et al. 2023), and our findings extend this conserved lipid‐based stress response to *L. minor* via ABA‐mediated signalling. The ability to rapidly switch TAG synthesis on and off in response to hormonal cues underscores the role of TAGs as dynamic storage lipids that buffer metabolic imbalances and provide flexibility during environmental fluctuations.

The physiological significance of ABA‐induced TAG accumulation could be further elucidated by using mutant lines in targeted physiological assays. However, such resources are currently limited for duckweeds, making this approach challenging at present. Future advances in knockout or knockdown techniques will help overcome these limitations and enable deeper investigation into the mechanisms and functional roles of lipid accumulation.

## 5 Conclusions

**Figure 7:**
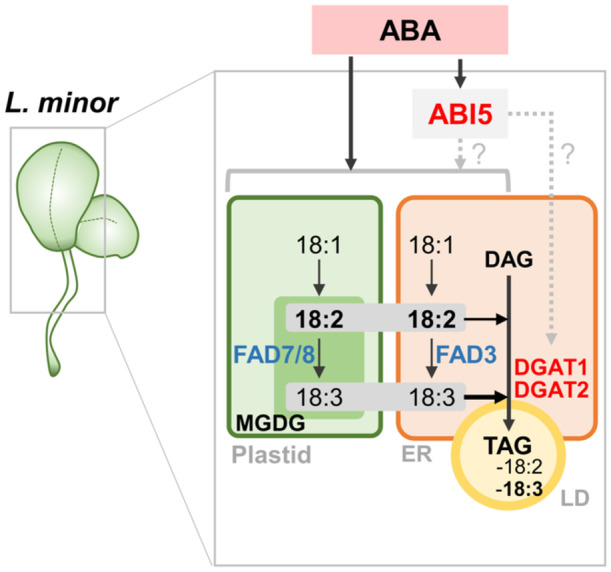
Proposed model of TAG accumulation in *L. minor* treated ABA. Environmental stresses such as salt stress might induce ABA accumulation, which in turn regulates lipid metabolism in *L. minor*. ABA signalling leads to the upregulation of *DGAT1* and *DGAT2*, which catalyse the final step of TAG biosynthesis from diacylglycerol (DAG) in the endoplasmic reticulum (ER). TAGs are stored in lipid droplets (LDs), and are enriched in polyunsaturated FAs such as 18:2 and 18:3. ABA treatment also represses the expression of desaturase genes *FAD3* and *FAD7/8*, which are responsible for the conversion of 18:2 to 18:3 in the plastid and ER, leading to a decrease in total 18:3 levels. The transcription factor *ABI5* is induced by ABA and may contribute to the transcriptional regulation of lipid metabolic genes. MGDG (monogalactosyldiacylglycerol), a chloroplast membrane lipid, serves as a potential source of polyunsaturated FAs for TAG synthesis under ABA‐induced stress. Arrows represent metabolic conversions or gene regulatory pathways; dashed arrows with question marks indicate hypothetical or unconfirmed regulatory links. [Color figure can be viewed at wileyonlinelibrary.com]

In conclusion, our study demonstrates that the phytohormone ABA, which is induced under abiotic stress conditions such as salt stress, triggers substantial TAG accumulation in *L. minor*, especially in chloroplast‐containing tissues. While ABA treatment inhibited growth of daughter fronds, it increased dry weight per frond, suggesting that carbon was redirected into storage molecules rather than growth. As illustrated in the model (Figure 7), ABA influences lipid biosynthetic enzymes and FA desaturation pathways. Specifically, ABA downregulated the expression of *FAD3* and *FAD7/8*, enzymes responsible for converting 18:2 to 18:3, leading to a decrease in 18:3 in tFAs. At the same time, it upregulated *DGAT1* and *DGAT2*, which catalyse the final step of TAG synthesis from DAG, resulting in enhanced TAG accumulation enriched in 18:2 and 18:3. The plastid‐derived MGDG pool, rich in polyunsaturated FAs, may serve as a substrate reservoir for TAG production, particularly under ABA‐triggered lipid remodelling. ABI transcription factors, such as *ABI5*, may contribute to the upregulation of *DGAT* and other TAG‐accumulation‐related genes. Taken together, our findings reveal a novel aspect of lipid metabolic regulation in *L. minor*, wherein ABA triggers coordinated plastid–ER lipid remodelling that results in selective TAG accumulation. Future studies should elucidate the molecular role of ABI5 and identify additional transcriptional regulators and/or lipases involved in MGDG degradation and lipid flux under ABA‐induced stress.

## Conflicts of Interest

The authors declare no conflicts of interest.

## Supporting information


**Figure S1:** Comparison of *L. minor* growth in 1/2 HG and 1/2 SH media. **Figure S2:** Lipid composition of *L. minor* under control, mock, and ABA treatments. **Figure S3:** Lipid composition of *L. minor* treated with various concentrations of ABA. **Figure S4:** Growth of *L. minor* fronds under ABA treatment. **Figure S5:** Phenotypic analysis of *L. minor* following ABA treatment and recovery. **Figure S6:** Lipid profiles in *L. minor* under salt stress conditions. **Figure S7:** Phenotype of *S. polyrhiza* treated with phytohormones.

Supporting Information Table Kim‐et‐al. **Table S1:** Primers used in this study. **Table S2:** Putative lipid related and ABA responsive genes in *L. minor*. **Table S3:** The gene ID of DGAT and fatty acid desaturase genes used in this study. **Table S4:** TargetP 2.0 subcellular localization prediction for fatty acid desaturases in *L. minor*. **Table S5:** Statistics for genome assessment using BUSCO (liliopsida).

## Data Availability

The data that support the findings of this study are openly available in figshare at https://doi.org/10.6084/m9.figshare.30143224.v1, reference number 30143224.v1.
