# Supplementary material for: Abscisic Acid Induces Triacylglycerol Accumulation and Lipid Remodelling in Chloroplast‐Containing Green Tissues of Lemna minor
Source: Plant Cell Environ. 2026 Jan 20;49(5):2464–79. doi: 10.1111/pce.70386 (PMC13107237; doi:10.1111/pce.70386)
Supplement: Supplementary file 1 — Figure S1: Comparison of L. minor growth in 1/2 HG and 1/2 SH media. Figure S2: Lipid composition of L. minor under control, mock, and ABA treatments. Figure S3: Lipid composition of L. minor treated with various concentrations of ABA. Figure S4: Growth of L. minor fronds under ABA treatment. Figure S5: Phenotypic analysis of L. minor following ABA treatment and recovery. Figure S6: Lipid profiles in L. minor under salt stress conditions. Figure S7: Phenotype of S. polyrhiza treated with phytohormones. [file PCE-49-2464-s001.docx]

## Supporting Information

**Abscisic acid induces triacylglycerol accumulation and lipid remodeling in chloroplast-containing green tissues of *Lemna minor***

Eunbi Kim, Bae Young Choi, Sujeong Je, Joohyun Kang, Seungwoo Shin, Yoomi Roh, Min Kim, Shogo Ito, Tokitaka Oyama, Yuree Lee, Donghwan Shim, Yasuyo Yamaoka


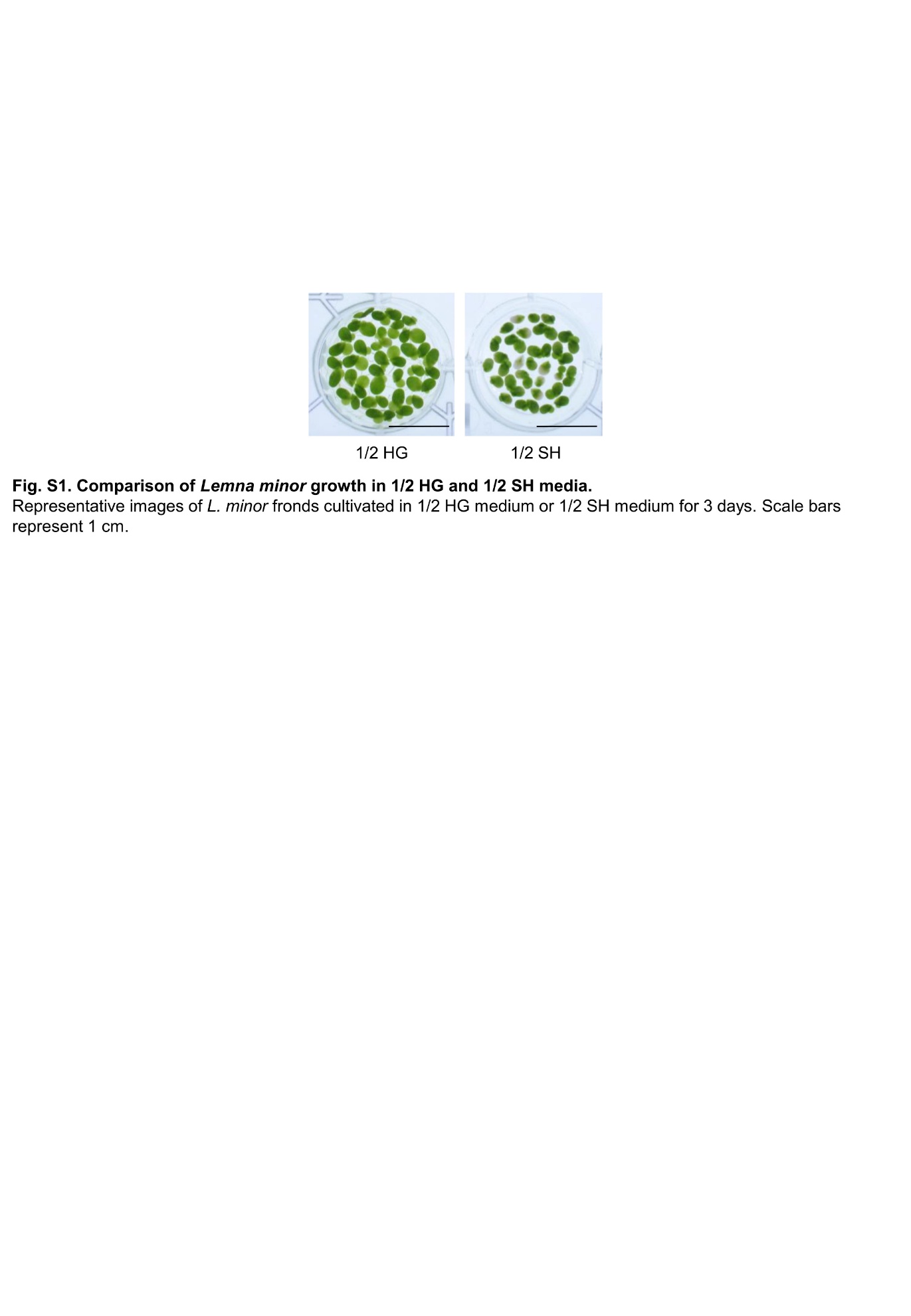


**Figure S1. Comparison of *L. minor* growth in 1/2 HG and 1/2 SH media**.
Representative images of *L. minor* fronds cultivated in 1/2 HG medium or 1/2 SH medium for 3 days. Scale bars represent 1 cm.


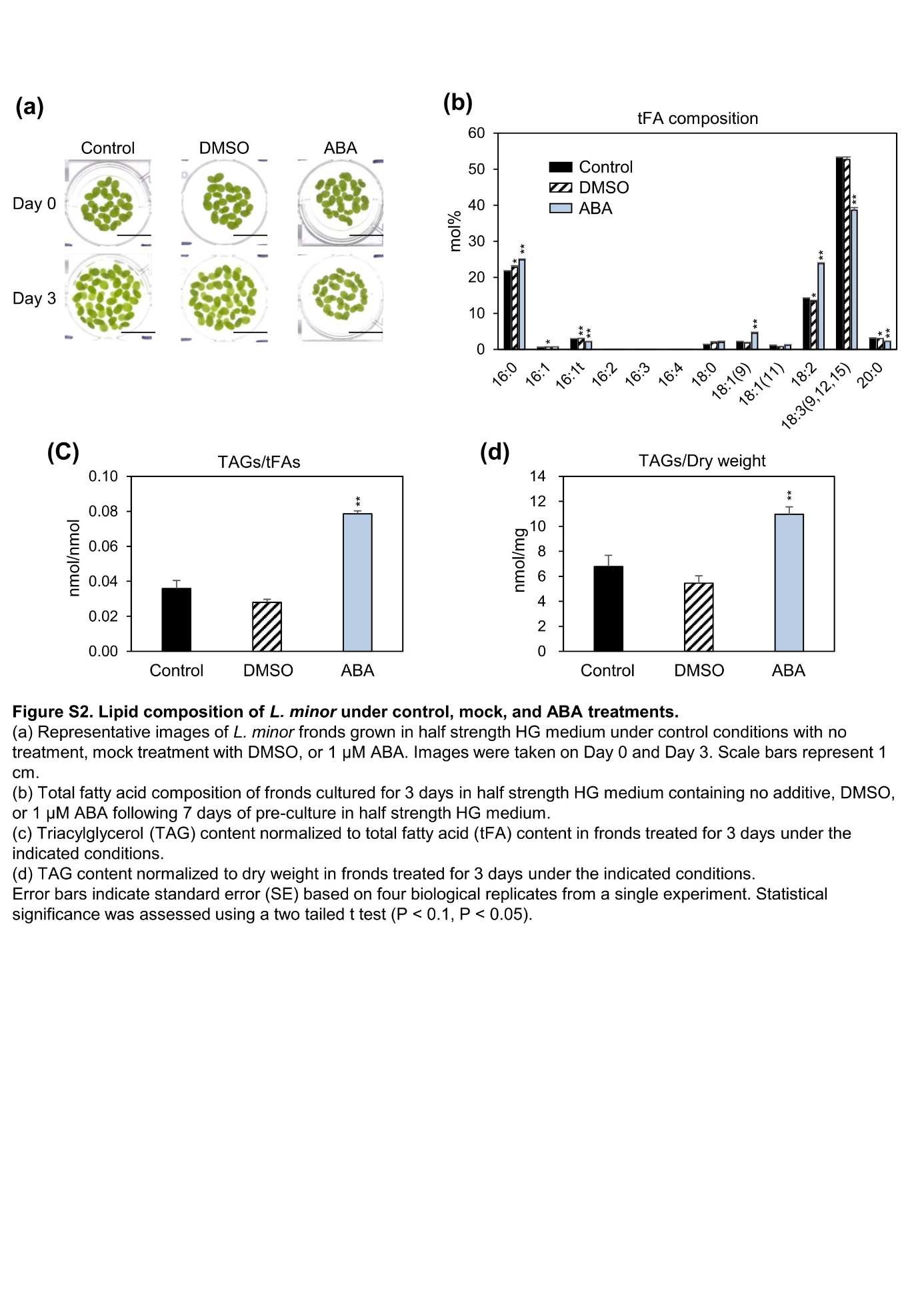


**Figure S2. Lipid composition of *L. minor* under control, mock, and ABA treatments.**

(a) Representative images of *L. minor* fronds grown in half strength HG medium under control conditions with no treatment, mock treatment with DMSO, or 1 μM ABA. Images were taken on Day 0 and Day 3. Scale bars represent 1 cm.

(b) Total fatty acid composition of fronds cultured for 3 days in half strength HG medium containing no additive, DMSO, or 1 μM ABA following 7 days of pre-culture in half strength HG medium.

(c) Triacylglycerol (TAG) content normalized to total fatty acid (tFA) content in fronds treated for 3 days under the indicated conditions.

(d) TAG content normalized to dry weight in fronds treated for 3 days under the indicated conditions.

Error bars indicate standard error (SE) based on four biological replicates from a single experiment. Statistical significance was assessed using a two tailed t test (**P* < 0.1, ***P* < 0.05).


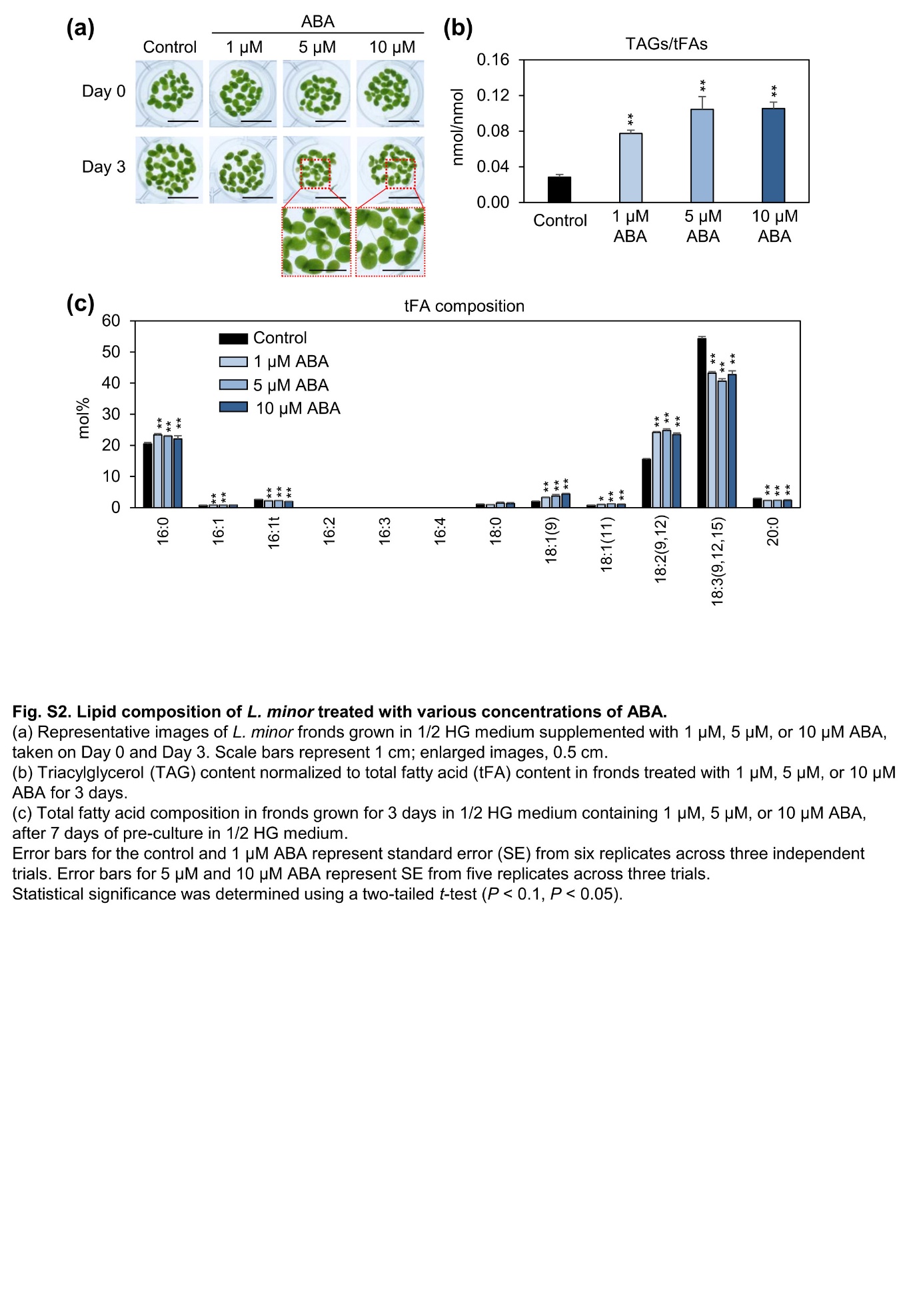


**Figure S3. Lipid composition of *L. minor* treated with various concentrations of ABA.**(a) Representative images of *L. minor* fronds grown in 1/2 HG medium supplemented with 1 μM, 5 μM, or 10 μM ABA, taken on Day 0 and Day 3. Scale bars represent 1 cm; enlarged images, 0.5 cm. (b) Triacylglycerol (TAG) content normalized to total fatty acid (tFA) content in fronds treated with 1 μM, 5 μM, or 10 μM ABA for 3 days. (c) Total FA composition in fronds grown for 3 days in 1/2 HG medium containing 1 μM, 5 μM, or 10 μM ABA, after 7 days of pre-culture in 1/2 HG medium. Error bars for the control and 1 μM ABA represent standard error (SE) from six replicates across three independent trials. Error bars for 5 μM and 10 μM ABA represent SE from five replicates across three trials. Statistical significance was determined using a two-tailed t-test (**P* < 0.1, ***P* < 0.05).


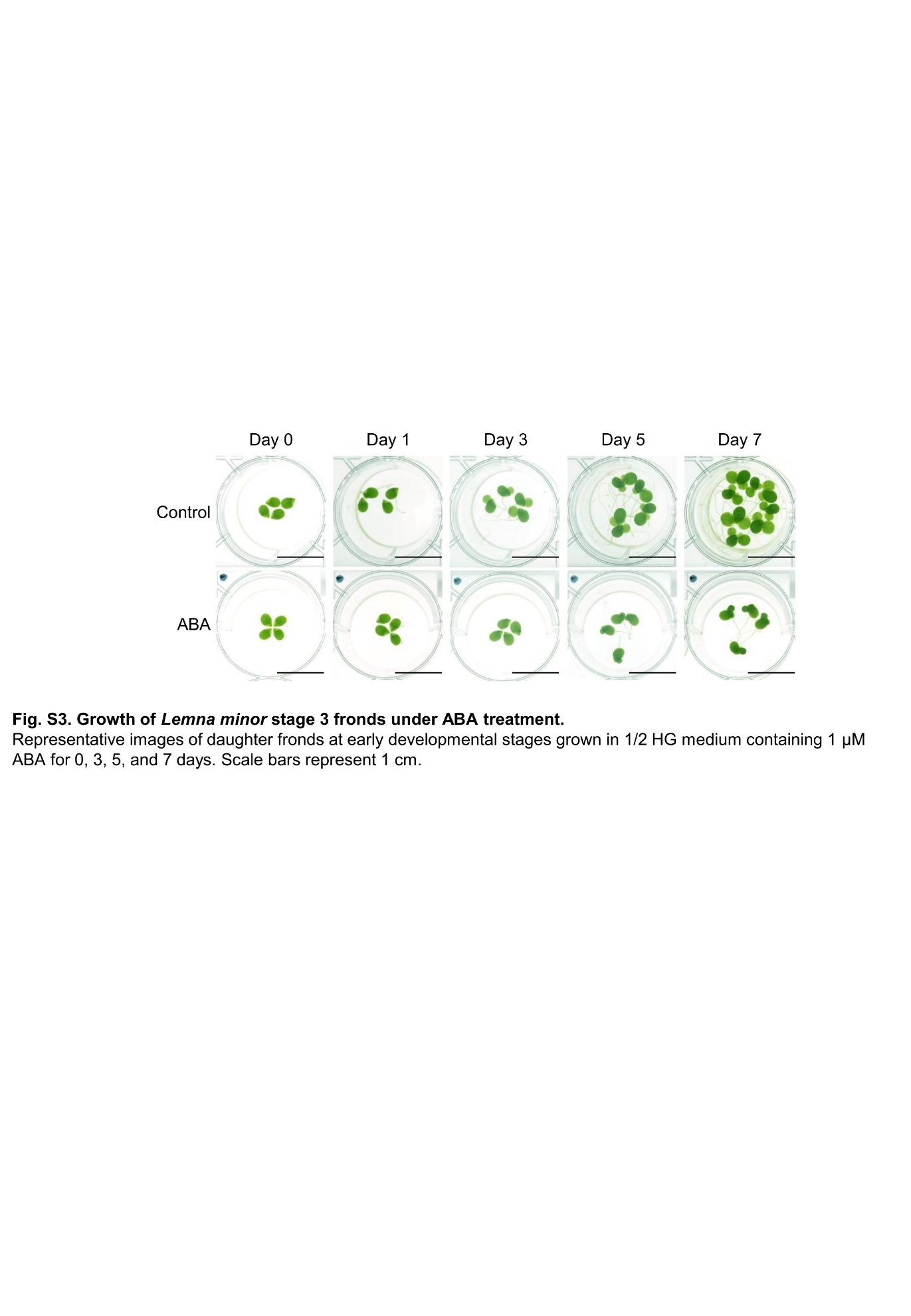


**Figure S4. Growth of *L. minor* fronds under ABA treatment.**Representative images of daughter fronds at early developmental stages grown in 1/2 HG medium containing 1 μM ABA for 0, 3, 5, and 7 days. Scale bars represent 1 cm.


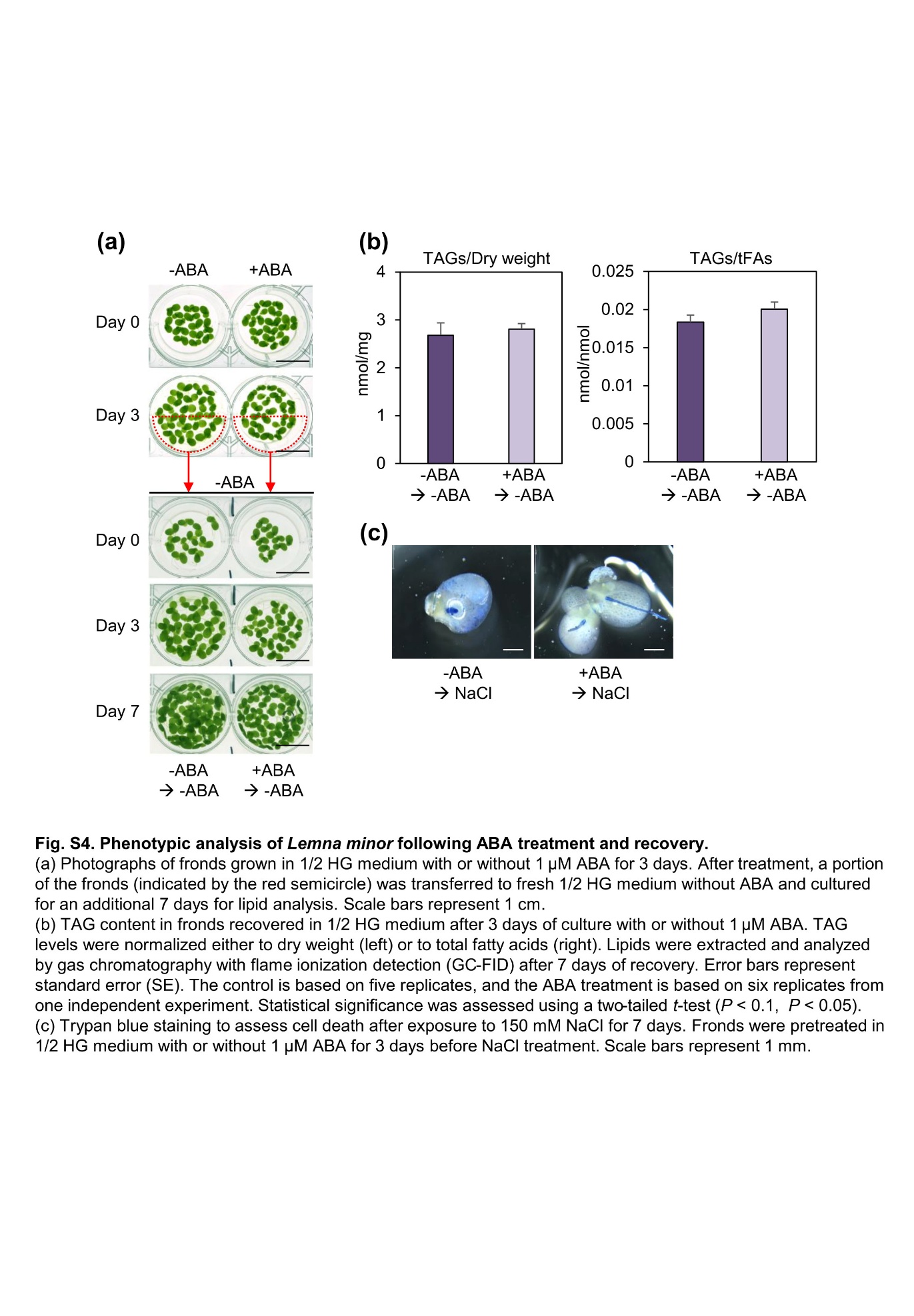


**Figure S5. Phenotypic analysis of *L. minor* following ABA treatment and recovery.**
(a) Photographs of fronds grown in 1/2 HG medium with or without 1 μM ABA for 3 days. After treatment, a portion of the fronds (indicated by the red semicircle) was transferred to fresh 1/2 HG medium without ABA and cultured for an additional 7 days for lipid analysis. Scale bars represent 1 cm.
(b) TAG content in fronds recovered in 1/2 HG medium after 3 days of culture with or without 1 μM ABA. TAG levels were normalized either to dry weight (left) or to total FAs (right). Lipids were extracted and analyzed by gas chromatography with flame ionization detection (GC-FID) after 7 days of recovery. Error bars represent standard error (SE). The control is based on five replicates, and the ABA treatment is based on six replicates from one independent experiment. Statistical significance was assessed using a two-tailed *t*-test (**P* < 0.1, ***P* < 0.05).
(c) Trypan blue staining to assess cell death after exposure to 150 mM NaCl for 7 days. Fronds were pretreated in 1/2 HG medium with or without 1 μM ABA for 3 days before NaCl treatment. Scale bars represent 1 mm.


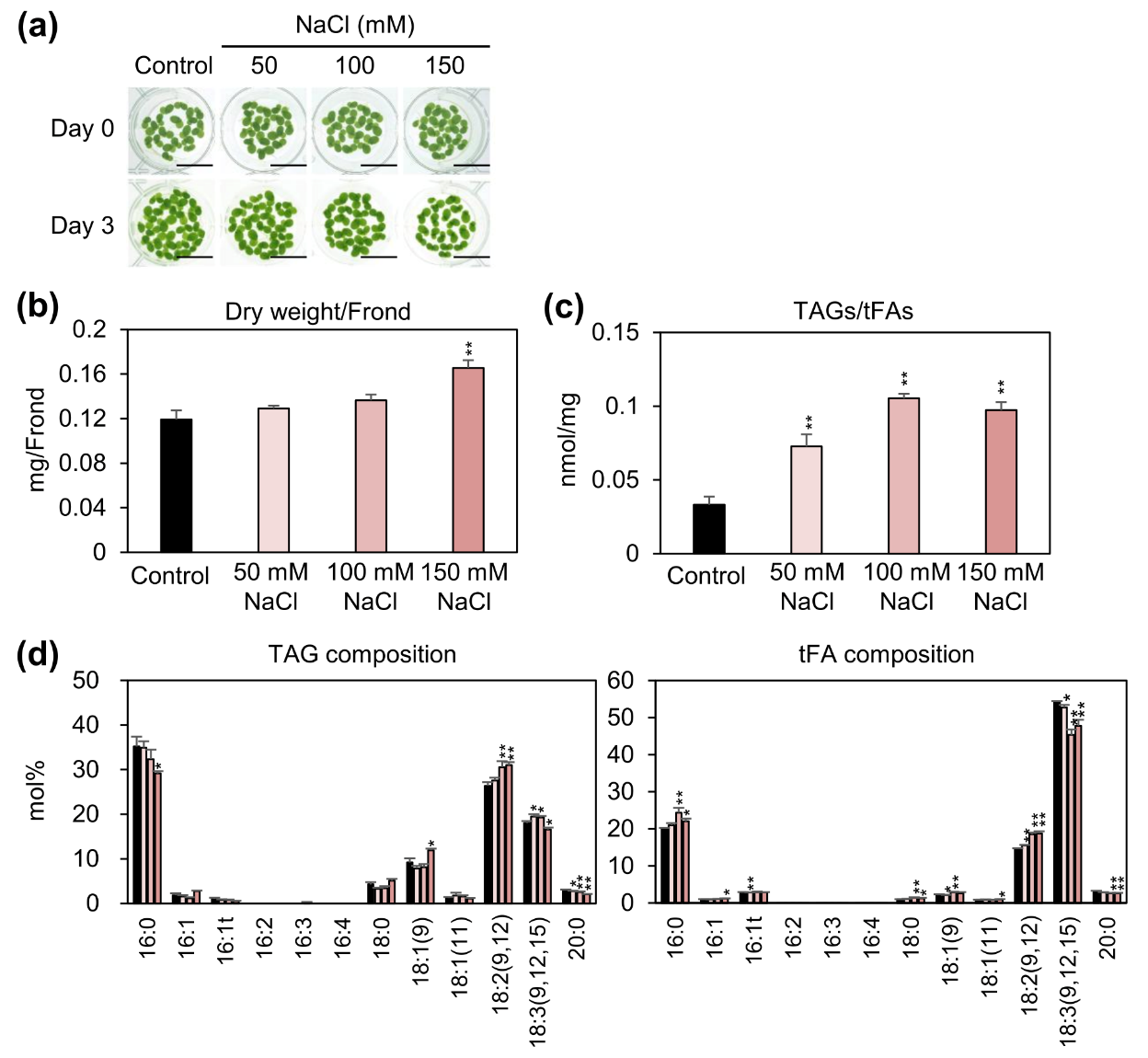


**Figure S6. Lipid profiles in *L. minor* under salt stress conditions.**

(a) Representative images of fronds grown in 1/2 HG medium containing various concentrations of NaCl (50, 100, or 150 mM). Photographs were taken at Day 0 and Day 3. Scale bars represent 1 cm.

(b) Dry weight per frond following 3 days of NaCl treatment. Values were normalized to the number of fronds. Error bars show standard error (SE) based on six biological replicates from three independent experiments.

(c) Triacylglycerol (TAG) content normalized to total fatty acids (tFAs) in fronds treated with NaCl. Error bars represent SE from four replicates across three independent experiments.

(d) FA composition of TAG and tFA fractions from fronds treated with 0 mM (control), 50 mM, 100 mM, or 150 mM NaCl for 3 days, following 7 days of pre-culture in 1/2 HG medium. For TAG composition, error bars indicate SE from four replicates across three independent experiments. For tFA composition, error bars represent SE from six replicates across three independent experiments.

Statistical significance was assessed using a two-tailed t-test (**P* < 0.1, ***P* < 0.05).


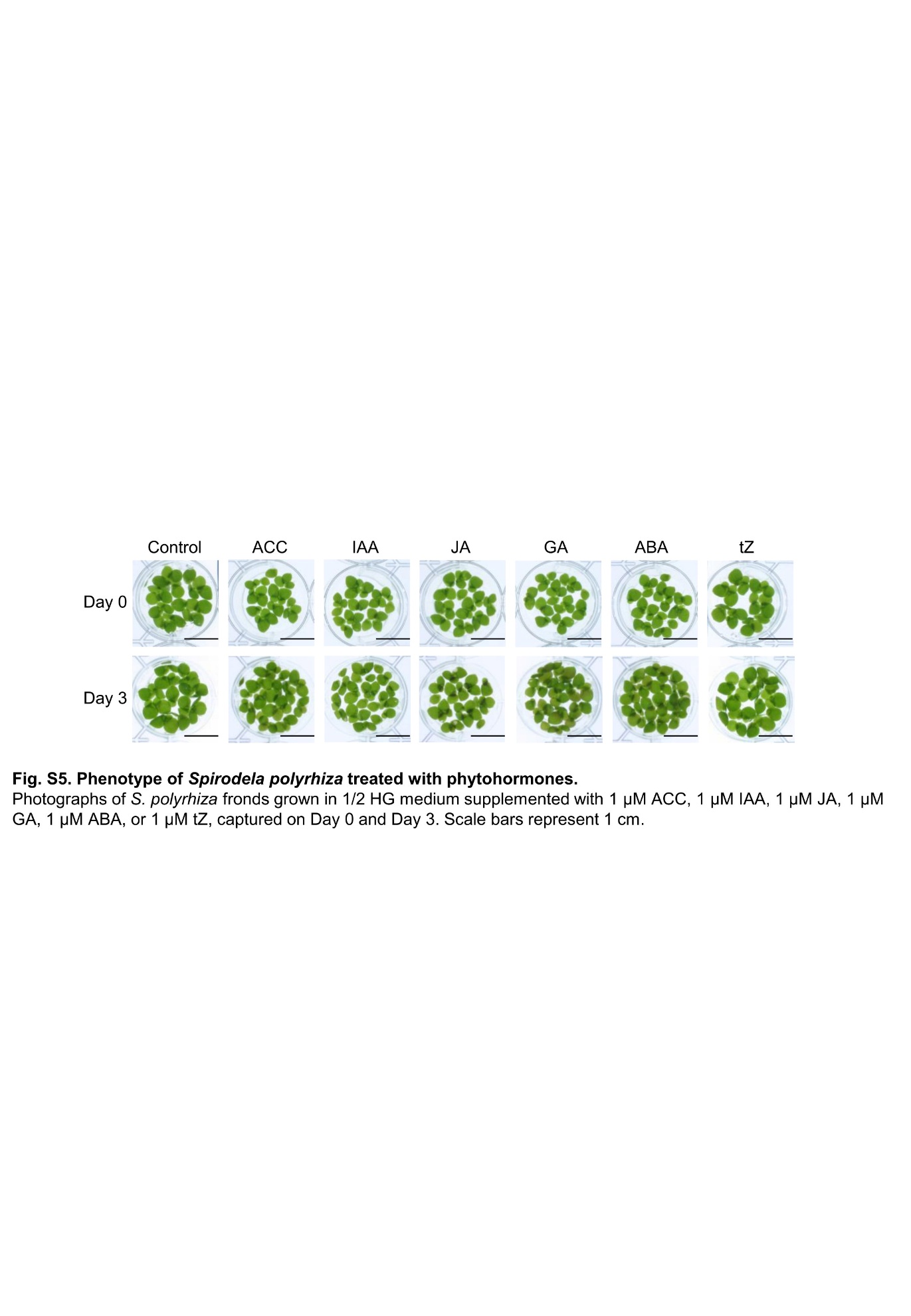


**Figure S7. Phenotype of *S. polyrhiza* treated with phytohormones.**

Photographs of *S. polyrhiza* fronds grown in 1/2 HG medium supplemented with 1 μM ACC, 1 μM IAA, 1 μM JA, 1 μM GA, 1 μM ABA, or 1 μM tZ, captured on Day 0 and Day 3. Scale bars represent 1 cm.
